# An increase in micro-vessels beneath the pleural surface on computed tomography as a preoperative predictor of pleural adhesions: a prospective study

**DOI:** 10.1007/s00595-025-03022-0

**Published:** 2025-02-26

**Authors:** Tomoyuki Kawamura, Naohiro Kobayashi, Takahiro Yanagihara, Yukinobu Goto, Hideo Ichimura, Yukio Sato

**Affiliations:** Department of Thoracic Surgery, University of Tsukuba Hospital, 2-1-1 Amakubo, Tsukuba, Ibaraki 305-8576 Japan

**Keywords:** Pleural adhesion, Preoperative CT, Blood flow, Thoracic surgery

## Abstract

**Purpose:**

Pleural adhesions, which may be present in varying degrees and involve blood vessels, often complicate thoracic surgery. The blood flow within pleural adhesions can be identified on computed tomography (CT) as micro-vessels beneath the pleural surface (MVBP). We conducted a prospective study to assess if MVBP can be detected on preoperative CT to predict intraoperative pleural adhesions.

**Methods:**

This prospective study investigated the preoperative CT images of patients scheduled to undergo surgery for lung tumors. MVBP was defined as positive when lung vessels were identified below the pleura on CT. MVBP was evaluated for each lung segment, and intraoperative findings of pleural adhesions on the segments were then recorded.

**Results:**

This study included 173 patients, with 1532 segments evaluated. Pleural adhesions were found in 51 patients and confirmed in 92 segments. The number of segments evaluated preoperatively as MVBP-positive was 134 (9%), of which 36 (26.9%) had pleural adhesions. Multivariable analysis identified that MVBP was an independent significant predictor (odds ratio = 2.29, 95% confidence interval 1.09–4.80, *P* = 0.028) of pleural adhesions on a per-patient basis.

**Conclusions:**

MVBP is a valuable predictor of pleural adhesions. The method is useful in clinical practice because it does not require additional examinations and is easy to assess.

## Introduction

Pleural adhesions are not present in the thoracic cavity originally, but are caused by previous inflammation of the pleura (such as pleuritis) and thoracic surgeries. Pleural adhesions make surgery difficult and may increase the risk of organ damage, greater blood loss, prolonged operative time, and the need for conversion to open chest surgery. Severe pleural adhesions have been reported to cause intraoperative pulmonary fistulas, prolong postoperative drainage, and extend the postoperative hospital stay [1–5]. There are various types of pleural adhesions, such as dense or loose adhesions. Dense pleural adhesions sometimes have blood vessels between the lung and chest wall (BVLC) [6]. Generally, pulmonary vessels become thinner as they approach the visceral pleura and CT cannot identify them just below the pleura. However, in the area of pleural adhesions with BVLC, increased blood flow beneath the pleura can be detected as pulmonary vessels extending beneath the pleura on CT. In our previous report, we defined these micro-vessels extending beneath the pleura as ‘micro-vessels beneath the pleural surface’ (MVBP) [6]. MVBP were assumed to be connected to BVLC within the pleural adhesions; therefore, the detection of MVBP would enable us to predict the presence of pleural adhesions. Previously, we reported a retrospective review of the presence of MVBP on CT in patients with pleural adhesions at surgery [6]. After pleural adhesion was identified in the operative record, the preoperative CT was reviewed retrospectively to investigate the presence of MVBP in the same area as the pleural adhesion. In the retrospective study, there were 56 cases of pleural adhesions with BVLC, of which 44 (79%) had MVBP on preoperative CT. Furthermore, 19 (90%) of 21 patients with BVLC thicker than 1 mm had MVBP; however, the study was retrospective and revealed only that preoperative CT findings might predict pleural adhesions. Therefore, in the present prospective cohort study, MVBP were retrieved from preoperative CT images to predict pleural adhesions. The purpose of the study was to assess the value of this new method of predicting pleural adhesions.

## Methods

**Fig. 1:**
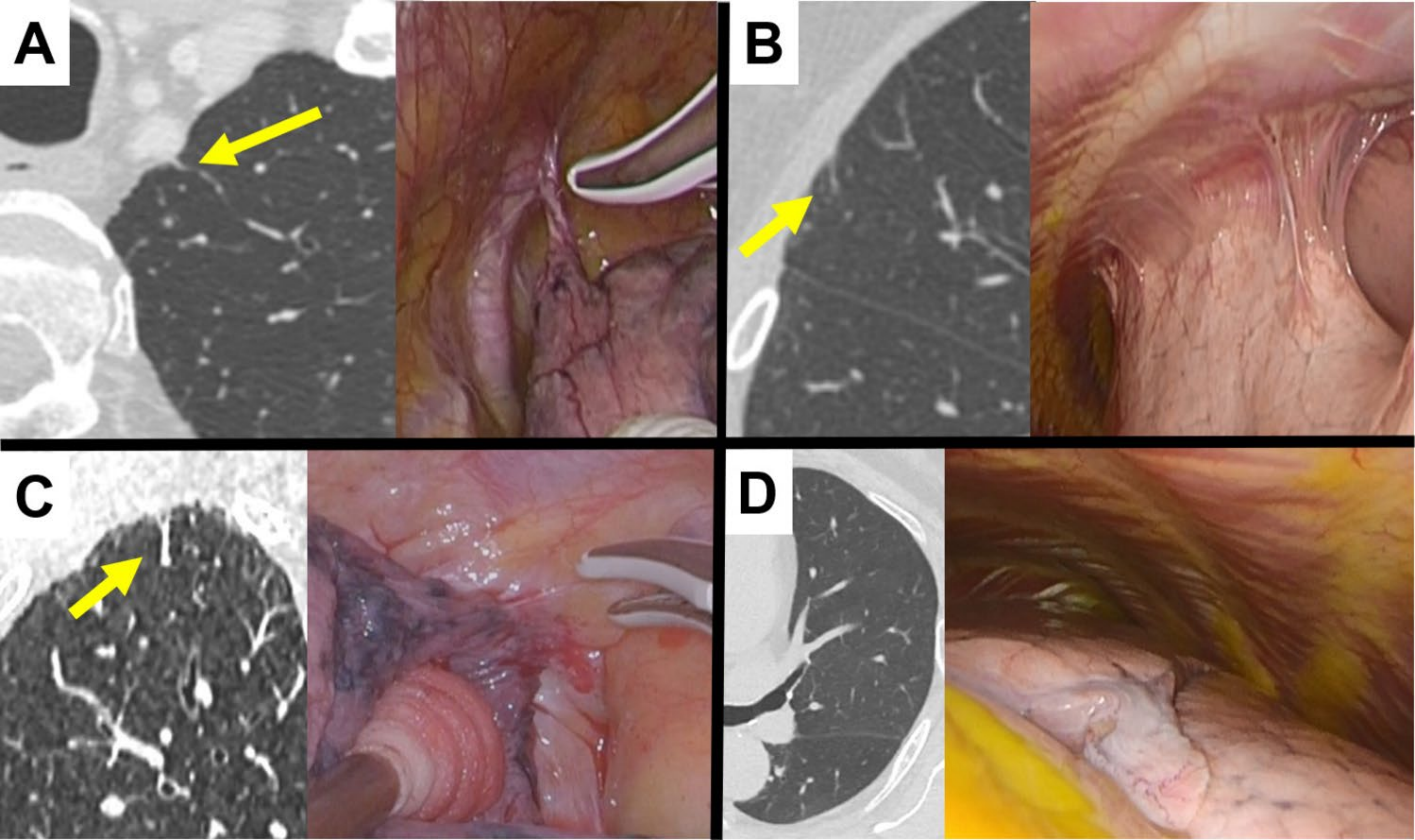
**A**–**C** Micro-vessels beneath the pleural surface (MVBP) were detected on preoperative computed tomography (CT), suggestive of the presence of pleural adhesions in these patients. At the time of surgery, pleural adhesions were confirmed to be present. **D** MVBP were undetectable on preoperative CT and the absence of pleural adhesions was suspected in this patient. At the time of surgery, pleural adhesions were confirmed to be absent. *MVBP* micro-vessels beneath the pleural surface

The subjects of this prospective observational study were patients who underwent lung resection for lung tumors (lung cancers, metastatic lung tumors, and benign lung tumors) at the University of Tsukuba Hospital between March, 2020 and March, 2021. The following patients were excluded from the study: those who had pneumothorax, pleuritis, or pyothorax within 3 months prior to surgery; those who underwent preoperative CT-guided needle biopsy; and those with chest wall invasion of the tumor. This study was not interventional and no new information or samples were obtained for the study. Approval was obtained from the Institutional Review Board of the University of Tsukuba Hospital (R01–241) and patients provided their informed consent using an opt-out method with a disclosure document. For this study, MVBP was defined as positive when lung vessels were identified just below the pleura on preoperative CT. MVBP were identified as a line leading to pulmonary vessels from the pleural surface to the pulmonary vessels. The other linear opacities can be distinguished by whether they are connected to pulmonary vessels. Figure 1 shows examples of MVBP findings and surgical findings. The CT used for evaluation was either plain or enhanced under a pulmonary window setting with a slice of 1–2 mm within 1 month before surgery, and coronal and sagittal images were also used in addition to axial images. MVBP were evaluated for each lung segment adjacent to the chest wall on the operative side to predict the area of discrepancies of pleural adhesions, with ten areas on the right side and eight areas on the left side. We excluded segments where the evaluation of MVBP was impossible because tumors were located beneath the pleura. MVBP were evaluated on the chest wall side, and not on the mediastinal or diaphragmatic sides. The MVBP were evaluated preoperatively by two thoracic surgeons. If the two evaluations differed, a decision was made after discussion. At the same time, the following factors that might be associated with pleural adhesions were recorded: past history of pneumonia, pleuritis/pyothorax, pneumothorax, thoracic surgeries, abdominal surgeries and chest injuries, comorbidity of interstitial pneumonitis and chronic obstructive pulmonary disease, chest X-ray finding of blunt costophrenic angle, CT findings of visceral pleural thickness, emphysematous and interstitial changes. The presence of pleural adhesion and BVLC was recorded for each segment in the surgical findings.

### Statistical analysis

This study was an exploratory study conducted to evaluate the new method of predicting pleural adhesions. The hypothesis was that MVBP are a predictor of pleural adhesions and was tested with a chi-square test for MVBP and pleural adhesions per lung segment. Then, sensitivity, specificity, positive predictive value (PPV), and negative predictive value (NPV) were calculated as measures of validity for predicting the presence of pleural adhesions and BVLC per lung segment. The important indicator for daily clinical practice use of this method was considered as “How often are pleural adhesions present when MVBP are detected on preoperative CT (PPV)?” or “How often are pleural adhesions absent when MVBP are not detected (NPV)?”. Therefore, the primary endpoint in this study was set to evaluate the accuracy of MVBP (calculated by PPV and NPV) to predict adhesions per lung segment.

To compare the prediction level of MVBP with that of other factors, sensitivity, specificity, PPV, and NPV were calculated per patient, and two logistic regression models were used for univariable and multivariable analyses. The variables with significance were entered into the multiple logistic regression models using the forward stepwise selection method. All statistical analyses were performed using IBM SPSS Statistics 24 (IBM, Inc., Armonk, NY). A difference was considered significant when the *P*-value was less than 0.05.

## Results

### Patient characteristics

Between March, 2020 and March, 2021, 174 patients underwent lung resection for lung tumors at the University of Tsukuba Hospital. One patient was excluded from the study because of chest wall invasion of the tumor. Table 1 lists the patients’ clinical characteristics. There were 115 men and 58 women, whose mean age at the time of surgery was 68.5 ± 9.2 years. There were 132 cases of primary lung cancer, 33 cases of metastatic lung tumors, 1 case of both primary lung cancer and metastatic lung tumor, and 7 cases of benign lung tumors. Ten patients had a recorded chest medical history on the ipsilateral side of the lung surgery site (pneumonia, *n* = 4; chest surgery, *n* = 4; chest injury, *n* = 2). No patient had a history of pleuritis or pyothorax. CT findings showing definite emphysematous change and interstitial change were observed in 31 and 17 patients, respectively, while a preoperative clinical diagnosis of chronic obstructive pulmonary disease and interstitial pneumonitis was made in 13 and 12 patients, respectively. A blunted costophrenic angle was seen on the chest X-ray of seven patients. Thoracoscopic surgery was performed in 131 patients, which was converted to open thoracotomy surgery for a bronchial injury in one patient. The surgical procedures were lobectomy (*n* = 105), segmentectomy (*n* = 27), wedge resection (*n* = 40), and biopsy (*n* = 1).

**Table 1:** Patients’ characteristics

	*n* = 173
Age, years (mean ± SD)	68.5 ± 9.2
Sex (male/female)	115/58
Smoking index (mean ± SD)	890 ± 600
Diagnosis (LC/Metastasis/LC and metastasis/benign tumor)	132/33/1/7
Past medical history (ipsilateral side of the surgery)	
Pneumonia/pleuritis or pyothorax/COPD/IP	6 (4)/0/13/12
Chest surgery/abdominal surgery/chest injury	22 (4)/65/2 (2)
CT findings (emphysematous changes/interstitial changes/pleural thickness)	31/17/16
Blunt costophrenic angle on chest X-ray	7
Surgical approach (VATS/RATS/open)	131/28/14
Procedure (lobectomy/segmentectomy/wedge/biopsy)	105/27/40/1
Tumor localization (RUL/RML/RLL/LUL/LLL)	61/ 5/36/45/25

*SD* standard deviation, *LC* lung cancer, *COPD* chronic obstructive pulmonary disease, *IP* interstitial pneumonia, *VATS* video-assisted thoracic surgery, *RATS* robot-assisted thoracic surgery, *RUL* right upper lobe, *RML* right middle lobe, *RLL* right lower lobe, *LUL* left upper lobe, *LLL* left lower lobe, *Wedge* wedge resection

### Evaluation of adhesion in each segment

The collective total of lung segments on the operative side was 1580 for the 173 patients, being 980 segments for 98 patients who underwent surgery on the right lung and 600 segments for 75 patients who underwent surgery on the left lung. CT findings of MVBP could not be evaluated because of tumors beneath the pleura in 47 segments, and because of severe peripheral lung changes influenced by previous lung wedge resection in one segment. As a result, MVBP findings were assessed for a total of 1532 segments. A cross-tabulation of MVBP on preoperative CT and pleural adhesions on surgical findings is shown on the left side of “Per lung segment” in Table 2. There were 134 (9%) segments evaluated as MVBP-positive and 1398 (91%) segments evaluated as MVBP-negative. The sensitivity, specificity, PPV, NPV, and accuracy were 39.1%, 93.2%, 26.9%, 96.0%, and 90.0% respectively. A Chi-square test result showed a significant relationship between MVBP and pleural adhesions (*P* < 0.001). A cross-tabulation of MVBP on preoperative CT and pleural adhesions on surgical findings is shown on the right side of “Per lung segment” in Table 2. The sensitivity, specificity, PPV, and NPV were 45.6%, 93.0%, 23.1%, and 97.4%, respectively. The sensitivity slightly increased by focusing on patients with BVLC in the pleural adhesions.

**Table 2:** Contingency table of micro-vessels beneath the pleural surface on preoperative computed tomography and pleural adhesions or blood vessels between the lung and chest wall on surgical findings

		Per lung segment				Per patient			
		Pleural adhesion		BVLC		Pleural adhesion		BVLC	
		+	−	+	−	+	−	+	−
MVBP	+	36	98	31	103	35	59	30	64
	−	56	1342	37	1361	15	63	12	66

*MVBP* micro-vessels beneath the pleural surface, *BVLC* blood vessels between lung and chest wall

### Predictive factors of pleural adhesions per patient

Table 3 shows the results of predictivity for each factor. On a per-patient basis, MVBP had a sensitivity of 70.0%, a specificity of 51.6%, a PPV of 37.2%, and an NPV of 80.8% for the prediction of pleural adhesions. Table 2 shows a cross-tabulation of MVBP on preoperative CT and pleural adhesions on surgical findings. Table 4 shows the two logistic regression models to predict pleural adhesions. In the univariable analysis, the odds ratio (OR) of MVBP was 2.16 (95% confidence interval [CI] 1.06–4.40,* P* = 0.035). The other predictors that showed significant differences in the univariable analysis were emphysematous change on CT (OR = 3.36, 95% CI 1.50–7.49, *P* = 0.031), interstitial change on CT (OR = 4.11, 95% CI 1.47–11.51, *P* = 0.007), and pleural thickness on CT (OR = 4.83, 95% CI 1.65–14.15, *P* = 0.004). Multivariable analysis revealed that the independent significant predictors were MVBP (OR = 2.29, 95% CI 1.09–4.80, *P* = 0.028), interstitial change on CT (OR = 4.64, 95% CI 1.60–13.48, *P* = 0.005), and pleural thickness on CT (OR = 4.71, 95% CI 1.56–14.20, *P* = 0.006).

**Table 3:** Sensitivity, specificity, positive predictive value, and negative predictive value of the predictive factors for pleural adhesions in each patient

	*n*	Sensitivity	Specificity	PPV	NPV
MVBP	94	70.0	51.6	37.2	80.8
Pleural thickness in CT findings	16	19.6	95.1	62.5	73.9
Blunt costophrenic angle on chest X-ray	7	5.9	96.7	42.9	71.1
Pneumonia	6	7.8	98.4	66.7	71.9
Ipsilateral side of the surgery	4	5.9	99.2	75.0	71.6
COPD	13	9.8	96.4	38.5	71.3
Emphysematous change on CT	31	31.4	87.7	51.6	75.4
Interstitial pneumonitis	12	13.7	95.9	58.3	72.7
Interstitial change on CT	17	19.6	94.3	58.8	73.7
Pneumothorax	2	3.9	100.0	100.0	71.3
Ipsilateral side of the surgery	1	2.0	100.0	100.0	70.9
Thoracic surgery	22	19.6	90.2	45.5	72.8
Ipsilateral side of the surgery	4	5.9	99.2	75.0	71.6
Abdominal surgery	65	39.2	63.1	30.8	71.3
Chest injury	2	19.6	90.2	45.5	72.8

*MVBP* micro-vessels beneath the pleural surface, *PPV* positive predictive value, *NPV* negative predictive value, *COPD* chronic obstructive pulmonary disease

**Table 4:** Univariable and multivariable logistic regression models of the predictive factors for pleural adhesions in each patient

	*n*	Univariable analyses		Multivariable analyses	
		OR (95% CI)	*P*-value	OR (95% CI)	*P*-value
MVBP	94	2.16 (1.06–4.40)	0.035	2.29 (1.09–4.80)	0.028
Pleural thickness on CT	16	4.83 (1.65–14.15)	0.004	4.71 (1.56–14.20)	0.006
Blunt costophrenic angle on chest X-ray	7	1.88 (0.41–8.74)	0.412		
Emphysematous change on CT	31	3.36 (1.50–7.49)	0.031		
Interstitial change on CT	17	4.11(1.47–11.51)	0.007	4.64 (1.6–13.48)	0.005
Pneumonia (ipsilateral side of the surgery)	4	7.72 (0.78–76.12)	0.079		
Thoracic surgery (ipsilateral side of the surgery)	4	5.04 (0.45–56.91)	0.191		

MVBP, micro-vessels beneath the pleural surface; OR, odds ratio; CI, confidence interval

## Discussion

In this prospective cohort study, we investigated whether MVBP could be a preoperative predictor of pleural adhesions. We found a significant relationship between MVBP and pleural adhesions, and that the PPV, NPV, and accuracy per lung segment were 26.9%, 96.0%, and 90.0%, respectively. Prediction by MVBP had a sensitivity of 70.0% per patient, which was higher than that of other predictors, and would be suitable for screening for pleural adhesions. MVBP is a CT finding of increased blood flow beneath the visceral pleura, with the micro-vessels connected to pulmonary vessels for perfusion. Therefore, MBVP can be easily distinguished from pleural thickening and fibrosis. In addition, pleural adhesions with BVLC have MVBP on preoperative CT near the adhesion site because of this pulmonary venous perfusion associated with increased blood flow beneath the visceral pleura. In a previous retrospective study, MVBP were detected near the pleural adhesion site on preoperative CT in 79% of patients with BVLC based on surgical findings [6]. Because MVBP could also enable prediction of the pleural adhesion site, in this study, MVBP were evaluated in each pulmonary segment to carry out the site-specific assessment. The PPV of MVBP for predicting pleural adhesions per area was 26.9% and the sensitivity was 39.1%. Moreover, the PPV of MVBP for predicting pleural adhesions with BVLC specifically, was 23.1% and the sensitivity was 45.6%. This means that a pleural adhesion exists in about a quarter of patients whose preoperative CT shows MVBP. PPV was relatively lower than other prediction factors and the false positive rate was high. This may be due to the fact that MVBP are caused not only by BVLC, but also as a result of the gravitational effect, partial pulmonary venous stagnation, and partially increased blood flow from old inflammatory changes.

There have been several studies on how to predict pleural adhesions [7–14]. Dynamic examinations such as ultrasonography, dynamic CT, and dynamic MRI to detect sliding of the visceral and parietal pleura during the inspiratory and expiratory phases are performed to help predict pleural adhesions. These tests have a relatively higher PPV than MVBP, with ultrasonography between 44 and 73% [7, 13] and dynamic CT at 39% [14]. However, pleural adhesions are not frequent (13–35%) [7, 13–15]. Therefore, performing these additional examinations for the sole purpose of predicting adhesions is controversial in daily clinical practice because of the burden on patients and medical staff, as well as the financial costs. Evaluation by MVBP is performed on conventional CT and involves no patient burden and low examiner effort. Other methods to predict pleural adhesions include evaluation of the blunted costophrenic angle on chest X-ray (PPV = 39%) or pleural thickening on CT (PPV = 58%) [15, 16]. Moreover, a medical history of inflammatory diseases such as pneumonia, pleuritis, and pyothorax, or a history of surgery on the ipsilateral side have also been recognized as predictors of adhesions. In this study, univariable analysis identified that MVBP, pleural thickness on CT, and interstitial change on CT were significant factors for predicting pleural adhesions. A history of pneumonia and thoracic surgery (ipsilateral side of the surgery) had relatively high odds ratios (7.72 and 5.04, respectively). Although not significant because of the small number of cases, these factors should be considered as strong predictors of pleural adhesions. Multivariable analysis identified that MVBP, pleural thickness on CT, and interstitial change on CT were independent factors. MVBP could be considered a predictor of pleural adhesions distinct from inflammatory change just below the pleura, as expressed by pleural thickness. Interstitial changes and pleural thickening have higher hazard ratios than MVBP. Therefore, pleural thickening and interstitial changes are more likely to be associated with pleural adhesions than MVBP.

This study had several limitations. First, pleural adhesions without BVLC cannot be predicted by MVBP. Second, in cases of extensive pleural adhesions, blood flow on the pleural surface is dispersed even with BVLC, and identification of MVBP would be difficult on CT. Therefore, it is difficult to predict pleural adhesions over the whole thoracic cavity, which are the most troublesome during thoracic surgery. Third, the detection of MVBP is difficult in patients with strong interstitial or emphysematous changes that make it hard to distinguish between pulmonary vessels and linear opacity. In this study, MVBP were evaluated by two thoracic surgeons. This is because MBVP are expected to be identified in daily clinical practice by the thoracic surgeons who will perform the surgery when reviewing the preoperative CT findings. However, a coincidence rate of the evaluation among observers has not been analyzed; therefore, reproducibility is an issue for the future.

The prediction of pleural adhesions by MVBP has the following benefits. Evaluation of MVBP by CT is simple and takes less than a minute, allowing the thoracic surgeon to check MVBP easily. Furthermore, the evaluation can be done with conventional preoperative CT and does not require additional examination or special abilities. Finally, it involves no extra economic burden on the patient or the medical system, and the burden on the evaluator is negligible. In contrast, the frequency of pleural adhesions is so low (13–35%) that the value of performing additional examinations just to predict pleural adhesions is questionable.

The detection of MVBP does not affect major treatment strategies. However, in the presence of MVBP on CT, pleural adhesions with BVLC could exist around the findings. Blood flow within the adhesion suggests dense adhesions and a careful approach to the thoracic cavity and an alteration of the skin incision site could reduce lung injury and bleeding. Moreover, if the presence of adhesions is known before surgery, it is possible to prepare for surgery associated with the removal of pleural adhesions; for example, by reserving more operating time and preparing the necessary surgical equipment. This study revealed the predictive ability of MVBP, although the PPV of 26.9% was relatively low. A combined assessment of MVBP and other predictors may be more valuable, especially when the pretest probability based on clinical findings is high.

## Conclusion

In this prospective study, the positive predictive value of pleural adhesions was 37.2% in the presence of MVBP on preoperative CT. The presence of MVBP is indicative of increased blood flow beneath the visceral pleura, which suggests that there might be pleural adhesions with blood vessels surrounding the MVBP (PPV = 23.1%).

## Data Availability

The data underlying this article will be shared upon reasonable request to the corresponding author.

## Abbreviations

BVLC: Blood vessels between lung and chest wall
CI: Confidence interval
MVBP: Micro-vessels beneath the pleural surface
NPV: Negative predictive value
OR: Odds ratio
PPV: Positive predictive value
